# Cyclin D1 expression in non-small-cell lung cancers: its association with altered p53 expression, cell proliferation and clinical outcome

**DOI:** 10.1038/sj.bjc.6990500

**Published:** 1999-06

**Authors:** T Mishina, H Dosaka-Akita, I Kinoshita, F Hommura, T Morikawa, H Katoh, Y Kawakami

**Affiliations:** First Department of Medicine and Second Department of Surgery, Hokkaido University School of Medicine, North 15, West 7, Sapporo, 060-8638, Japan

**Keywords:** cyclin D1, p53, p16/RB pathway, Ki-67 index, clinical outcome, non-small-cell lung cancers

## Abstract

Cyclin D1, like p16^INK4^ (p16) and retinoblastoma (RB) proteins, participates in the cell cycle control at the G1–S transition. We have previously demonstrated altered p16 and RB protein status in non-small-cell lung cancers (NSCLCs) and their potential synergistic effect with altered p53 protein on proliferative activity (Kinoshita et al (1996) *Cancer Res***56**: 5557–5562). In the present study, cyclin D1 expression was studied by immunohistochemistry in the same cohort of 111 resected NSCLCs as in our previous study, and the amount of the cyclin D1 gene was analysed by Southern blot analysis in 29 NSCLCs. Cyclin D1 expression was analysed in relation to the status of p53, p16 and RB proteins, and proliferative activity determined by the Ki-67 index. It was also analysed in relation to survival of 77 patients with NSCLCs which were potentially curatively resected between 1990 and 1995. We found that: (1) cyclin D1 was expressed in 13 (11.7%) of 111 NSCLCs; (2) the cyclin D1 gene was neither significantly amplified nor rearranged; (3) cyclin D1 expression significantly correlated with altered p53 protein expression (*P* = 0.04), whereas it did not correlate with p16 and RB protein status; (4) proliferative activity tended to be higher in cyclin D1-positive (+) tumours than in cyclin D1-negative (–) tumours, although this difference was not statistically significant (*P* = 0.08); and (5) patients with cyclin D1+ tumours survived longer than patients with cyclin D1– tumours (5-year survival rates, 89% and 64% respectively, by the Kaplan–Meier method; *P* = 0.045 by the log-rank test), and cyclin D1 expression tended to be a favourable prognostic factor (*P* = 0.08 in univariate analysis). These findings suggest the involvement of cyclin D1 in the development and progression of NSCLCs, their proliferative activity and clinical outcome of NSCLC patients. © 1999 Cancer Research Campaign


*British Journal of Cancer* (1999) **80**(8), 1289–1295
© 1999 Cancer Research Campaign
Article no. bjoc.1998.0500

### Cyclin D1 expression in non-small-cell lung cancers: its association with altered p53 expression, cell proliferation and clinical outcome

**T Mishina[1], H Dosaka-Akita[1], I Kinoshita[1], F Hommura[1], T Morikawa[2], H Katoh[2] and Y Kawakami[1]**

[1]First Department of Medicine and [2]Second Department of Surgery, Hokkaido University School of Medicine, North 15, West 7, Kita-ku, Sapporo, 060-8638, Japan

**Summary** Cyclin D1, like p16$^{INK4}$ (p16) and retinoblastoma (RB) proteins, participates in the cell cycle control at the G1–S transition. We have previously demonstrated altered p16 and RB protein status in non-small-cell lung cancers (NSCLCs) and their potential synergistic effect with altered p53 protein on proliferative activity (Kinoshita et al (1996) *Cancer Res* **56**: 5557–5562). In the present study, cyclin D1 expression was studied by immunohistochemistry in the same cohort of 111 resected NSCLCs as in our previous study, and the amount of the cyclin D1 gene was analysed by Southern blot analysis in 29 NSCLCs. Cyclin D1 expression was analysed in relation to the status of p53, p16 and RB proteins, and proliferative activity determined by the Ki-67 index. It was also analysed in relation to survival of 77 patients with NSCLCs which were potentially curatively resected between 1990 and 1995. We found that: (1) cyclin D1 was expressed in 13 (11.7%) of 111 NSCLCs; (2) the cyclin D1 gene was neither significantly amplified nor rearranged; (3) cyclin D1 expression significantly correlated with altered p53 protein expression ($P$ = 0.04), whereas it did not correlate with p16 and RB protein status; (4) proliferative activity tended to be higher in cyclin D1-positive (+) tumours than in cyclin D1-negative (–) tumours, although this difference was not statistically significant ($P$ = 0.08); and (5) patients with cyclin D1+ tumours survived longer than patients with cyclin D1– tumours (5-year survival rates, 89% and 64% respectively, by the Kaplan–Meier method; $P$ = 0.045 by the log-rank test), and cyclin D1 expression tended to be a favourable prognostic factor ($P$ = 0.08 in univariate analysis). These findings suggest the involvement of cyclin D1 in the development and progression of NSCLCs, their proliferative activity and clinical outcome of NSCLC patients.

**Keywords:** cyclin D1; p53; p16/RB pathway; Ki-67 index; clinical outcome; non-small-cell lung cancers

Cyclin D1 is a member of the G1 cyclin family involved in the regulation of the G1–S transition of the cell cycle (Matsushime et al, 1991; Motokura et al, 1991), perhaps the most important checkpoint in the mammalian cell cycle (Weinberg, 1995; Sherr, 1996). It mediates the phosphorylation and functional inactivation of retinoblastoma (RB) protein in association with the cyclin-dependent kinases CDK4 and CDK6. Hyperphosphorylation of RB protein leads to its dissociation from transcription factors such as E2F-1 that transcriptionally regulate growth-promoting genes. p16$^{INK4}$ (p16) protein inhibits CDK4- and CDK6-mediated phosphorylation of RB protein (Serrano et al, 1993; Hannon et al, 1994). We have previously demonstrated altered p16 and RB protein status in non-small-cell lung cancers (NSCLCs) and their potential synergistic effect with altered p53 protein on proliferative activity (Kinoshita et al, 1996). On the other hand, amplification and/or increased expression of the cyclin D1 gene have been reported in several kinds of human cancer, including those derived from the oesophagus, head and neck, liver, and breast, with prognostic importance in most of these cancers (Nishida et al, 1994; McIntosh et al, 1995; Naitoh et al, 1995; Åkervall et al, 1997). The association of cyclin D1 with cancer has led to the investigation of its oncogenic properties in vitro and in vivo. Cyclin D1 can either cooperate with the *ras* oncogene (Lovec et al, 1994) or complement a defective adenovirus E1a oncogene (Hinds et al, 1994) to transform cultured cells. In addition, targeting of the cyclin D1 gene by the MMTV promoter and by an Epstein–Barr virus promoter has been shown to result in mammary hyperplasia and carcinomas (Wang et al, 1994) and in premalignant dysplasia of the tongue, oesophagus and forestomach (Nakagawa et al, 1997) respectively, in transgenic mice. However, little is known about the cell–biological and clinical implications of cyclin D1 in NSCLCs, although its expression has been reported in resected NSCLCs (Shapiro et al, 1995; Betticher et al, 1996, 1997; Mate et al, 1996; Nishio et al, 1997; Marchetti et al, 1998; Tanaka et al, 1998).

In the present study, we examined cyclin D1 expression by immunohistochemistry in the same cohort of resected NSCLCs as in our previous study (Kinoshita et al, 1996), and the amount of the cyclin D1 gene by Southern blot analysis in a subset of tumours. Cyclin D1 expression was analysed in relation to the status of p53, p16 and RB proteins, to the proliferative activity determined by the Ki-67 index, and to clinical outcome.

### MATERIALS AND METHODS

### Tumour specimens and survival data

Primary tumour specimens from 111 NSCLCs were obtained by surgery from the Hokkaido University Medical Hospital, Sapporo Minami-Ichijo Hospital and National Sapporo Minami Hospital during 1990 and 1995. Tumours were snap-frozen in liquid nitrogen and stored at –80°C in OCT compound (Miles, Elkhart, IN, USA) for immunohistochemistry. For 29 specimens, portions of the tumour were also snap-frozen and stored at –80°C for

*Received 26 August 1998*
*Revised 22 December 1998*
*Accepted 22 December 1998*

*Correspondence to:* H Dosaka-Akita genomic DNA preparation. Adjacent non-frozen blocks were fixed in 10% neutral buffered formalin and embedded in paraffin, and then several sections from each specimen were stained with haematoxylin and eosin to observe histopathology. According to the 1981 WHO classification (WHO, 1982), they were histopathologically diagnosed as adenocarcinoma ($n$ = 71), squamous cell carcinoma ($n$ = 34) and large cell carcinoma ($n$ = 6). The post-surgical pathologic tumour, node, metastasis stage (pTNM) was determined according to the guidelines of the American Joint Committee on Cancer (Beahrs et al, 1992).

Of 111 NSCLCs, 92 tumours were potentially curatively resected. Of the 92 patients, survival was analysed for the 77 patients who met the following criteria: (1) they survived for more than 3 months after surgery; (2) did not die of causes other than lung cancer within 5 years after surgery; and (3) were followed up for more than 2 years after surgery (for patients who remained alive). Eleven patients who did not meet the above criteria (two died within 3 months after surgery, five died of causes other than lung cancer within 5 years after surgery, four were followed up for no more than 2 years after surgery), and four patients for whom no survival records after surgery were obtained were excluded from the present study. Tumour specimens were histopathologically diagnosed as adenocarcinoma ($n$ = 50), squamous cell carcinoma ($n$ = 22) and large cell carcinoma ($n$ = 5). They represented 52 stage I, nine stage II and 16 Stage IIIA tumours. Twenty-four patients received combination chemotherapy as post-surgical treatment. Radiation therapy was not performed before or after surgery for any patients. Because all the patients enrolled in the current study were coded, they could not be individually identified.

### Immunohistochemistry for cyclin D1

Five-micrometer frozen sections of NSCLC specimens were fixed in 3.7% formaldehyde in phosphate-buffered saline (PBS) (pH 7.2) at room temperature for 10 min, and microwave pretreatment in 10 mM citrate buffer (pH 6.0) was performed for 10 min to retrieve the antigenicity. The sections were then immersed in methanol containing 0.6% hydrogen peroxide for 20 min to block the endogenous peroxide activity, and were incubated with normal rabbit serum to block the non-specific antibody binding sites. The sections were consecutively reacted with mouse monoclonal anti-human cyclin D1 antibody DCS-6 (Bartkova et al, 1994; Lukas et al, 1994*a*) (Oncogene Science, Inc., Manhasset, NY, USA) or with control mouse isotype-specific immunoglobulin at 4°C overnight. Immunostaining was performed by the biotin–streptavidin immunoperoxidase method with 3,3′-diaminobenzidine as a chromogen (SAB-PO kit; Nichirei, Tokyo, Japan). Methyl green was used for counterstain. An oesophageal cancer specimen with cyclin D1 expression (courteously provided by Dr M Fujita of the Department of Pathology, National Sapporo Hospital, Sapporo, Japan), was used as a positive control each time. Tumours were regarded as cyclin D1-positive (+) if at least more than several malignant cells had nuclear staining by observing one whole specimen from each tumour. Tumours were scored as cyclin D1-negative (–) if all malignant cells had no nuclear staining. All immunohistochemical studies were done without knowledge of the clinical data.

### Southern blot analysis

Genomic DNA was extracted from 29 NSCLC tumour specimens, 25 of which were studied for cyclin D1 expression by immunohistochemistry. Southern blotting was carried out after digestion with *Eco*RI, and hybridized to [$\alpha$-$^{32}$P]dCTP-labelled human cyclin D1 cDNA (kindly provided by Dr A Arnold, Massachusetts General Hospital, Boston, MA, USA) and cardiac actin cDNA (kindly provided by Dr F Gunning, Stanford University and Veterans Affairs Medical Center, Palo Alto, CA, USA). The final wash was performed twice in 0.2 × saline–sodium citrate (SSC), 0.1% sodium dodecyl sulphate (SDS) at 50°C for 20 min. The blots were then exposed to Kodak XAR-5 film (Kodak, Rochester, NY, USA) and quantitatively analysed using a densitometer. The autoradiographs were developed at various times to ensure that this analysis was performed in the linear range of the film. The cyclin D1 signal intensity was normalized against the cardiac actin signal intensity. The relative cyclin D1 gene amount was defined as the normalized cyclin D1 signal intensity in the tumour samples relative to that in the normal lung control (= 1.0). The cyclin D1 gene was considered to be amplified when the relative cyclin D1 gene amount was more than 3.0, excluding the possibility of chromosomal aneuploidy.

### Ki-67 staining and immunohistochemistry for p53, p16 and RB proteins

For Ki-67 staining, and immunohistochemistry for p53, p16 and RB proteins, the slides and results that had been previously reported (Kinoshita et al, 1996) were used for the current study. All immunohistochemical studies were done without knowledge of the clinical data.

**Table 1** Relationship between cyclin D1 expression and various characteristics in 111 resected NSCLCs

| | Cyclin D1 expression | | |
|---|---|---|---|
| **Characteristic** | **(–)** | **(+)** | ***P*** |
| Age (years, mean ± s.d.) | 63.3 ± 9.0 | 64.1 ± 12.1 | 0.8 |
| Gender | | | |
| Male | 72 | 9 | 0.7 |
| Female | 26 | 4 | |
| Smoking (pack years) | | | |
| 0–19 | 27 | 6 | 0.2 |
| ≥ 20 | 71 | 7 | |
| Histological type[a] | | | |
| Adeno | 62 | 9 | 0.7 |
| Squamous | 30 | 4 | |
| Large | 6 | 0 | |
| pT classification | | | |
| T1 | 30 | 1 | 0.1 |
| T2–4 | 68 | 12 | |
| pN classification | | | |
| N0 | 61 | 9 | 0.5 |
| N1–3 | 36 | 3 | |
| pM classification | | | |
| M0 | 91 | 12 | 1.0 |
| M1 | 7 | 1 | |
| pStage | | | |
| I | 52 | 8 | 0.8 |
| II | 11 | 0 | |
| IIIa | 23 | 3 | |
| IIIb | 6 | 1 | |
| IV | 6 | 1 | |

[a]Adeno, adenocarcinoma; Squamous, squamous cell carcinoma; Large, large cell carcinoma.

*© 1999 Cancer Research Campaign*

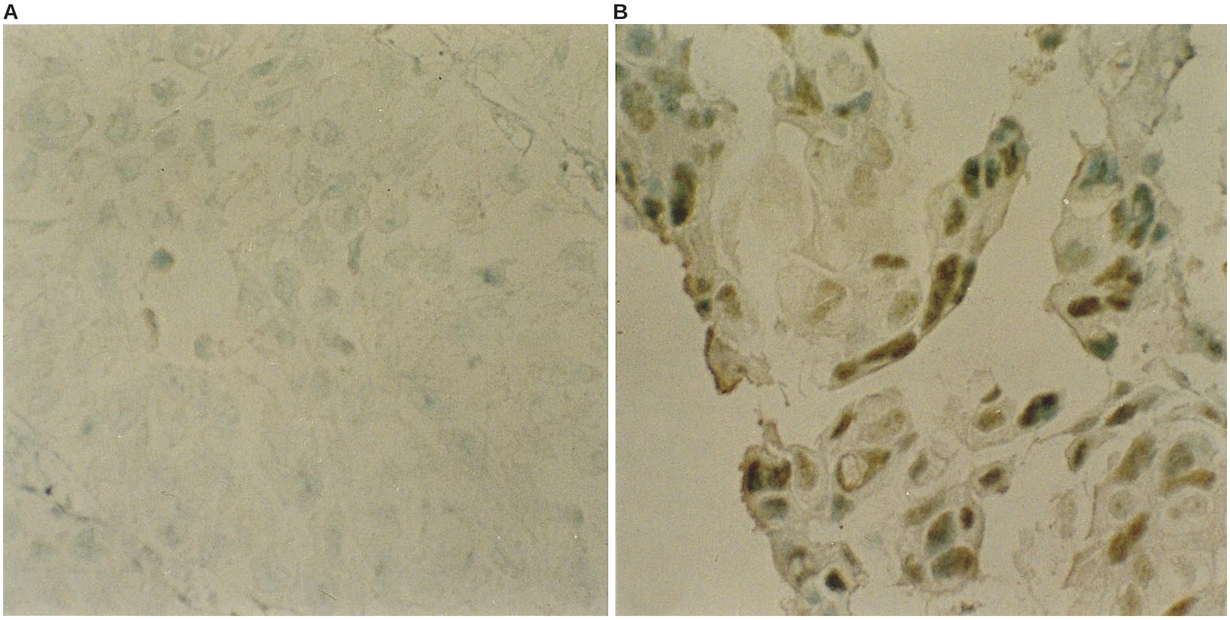

**Figure 1** Immunohistochemical staining patterns for cyclin D1 in primary NSCLCs. Representative cyclin D1-negative large-cell carcinoma displays no nuclear staining of cyclin D1 (**A**). Representative cyclin D1-positive adenocarcinoma shows moderate nuclear staining of cyclin D1 (**B**)

### Statistical analysis

The associations between cyclin D1 expression and categorical variables were analysed by the $\chi^2$ test or Fisher's exact test as appropriate. The associations between cyclin D1 expression and age were analysed by Student's *t*-test. The differences of Ki-67 indices between two groups were determined by the Mann–Whitney *U*-test. The survival curves were estimated using the Kaplan–Meier method, and differences in survival distributions were evaluated by the log-rank test. Cox's proportional hazards modelling of factors potentially related to survival was performed to identify which factors might have a significant influence on survival. The significance level chosen was $P < 0.05$, and all tests were two-sided.

### RESULTS

To determine cyclin D1 expression in tumour cells of NSCLCs, we subjected fresh-frozen tissues from 111 resected NSCLCs to immunohistochemistry for the protein. Cyclin D1 was expressed in 13 (11.7%) of 111 NSCLCs. Typical cyclin D1+ and cyclin D1– tumours are shown in Figure 1. All cyclin D1+ tumours had clear but weak to moderate nuclear staining, which was confined to a part of the tumour. Cytoplasmic staining was not observed in any tumour cells. On the other hand, normal epithelial cells, tumour stroma cells, and accompanying inflammatory cells were negative for cyclin D1 expression.

To explore the mechanisms of cyclin D1 expression, Southern blot analysis of the cyclin D1 gene was performed in 29 resected NSCLCs, since amplification of this gene is implicated in its expression in various types of tumours (Jiang et al, 1992; Buckley et al, 1993). However, the cyclin D1 gene was not significantly amplified and no rearranged bands were found in the 29 NSCLCs examined (Figure 2).

After completion of immunohistochemical analysis of cyclin D1, the status of this protein was statistically analysed to correlate it with clinical, clinicopathological and molecular biological characteristics. Cyclin D1 expression was found more frequently in tumours with advanced pT status than in pT1 tumours (12 of 80 pT2–4 tumours vs only one of 30 pT1 tumours), but this association was not statistically significant ($P = 0.1$) (Table 1). No other significant association between cyclin D1 expression and other clinical or clinicopathological parameters was observed.

Since cyclin D1 has been shown to be involved in the regulation of the G1–S transition of the cell cycle (Weinberg, 1995; Sherr, 1996), cyclin D1 expression was analysed in relation to the proliferative activity of tumours determined by the Ki-67 index, and to the status of p53, p16 and RB proteins involved in the G1–S transition control. The Ki-67 index tended to be higher in cyclin D1+ tumours than in cyclin D1– tumours, although this difference was not statistically significant ($P = 0.08$) (Table 2). Cyclin D1 expression was found significantly more frequently in tumours with altered p53 protein expression compared to tumours without it ($P = 0.04$), whereas it did not correlate with p16 or RB protein status (Table 2). Because p16 and RB proteins are indicated to function in a single regulatory pathway of the cell cycle (Serrano et al, 1993; Lukas et al, 1995) and are reciprocally lost in NSCLCs (Shapiro et al, 1995; Kinoshita et al, 1996), we divided the tumours into two groups, the tumours retaining both p16 and RB protein expression (p16+ and RB+; p16/RB+) and the tumours lacking either p16 or RB protein expression (p16– or RB–; p16/RB–). The difference in cyclin D1 expression between the two groups of tumours was not statistically significant (Table 2).

Finally, we analysed the relationship between cyclin D1 expression and post-surgical survival for 77 patients whose tumours were potentially curatively resected. Patients with cyclin D1+ tumours survived longer than patients with cyclin D1– tumours (5-year survival rates, 89% and 64% respectively; $P = 0.045$) (Figure 3), and cyclin D1 expression tended to be a favourable prognostic factor in univariate analysis ($P = 0.08$) (Table 3).

*© 1999 Cancer Research Campaign*

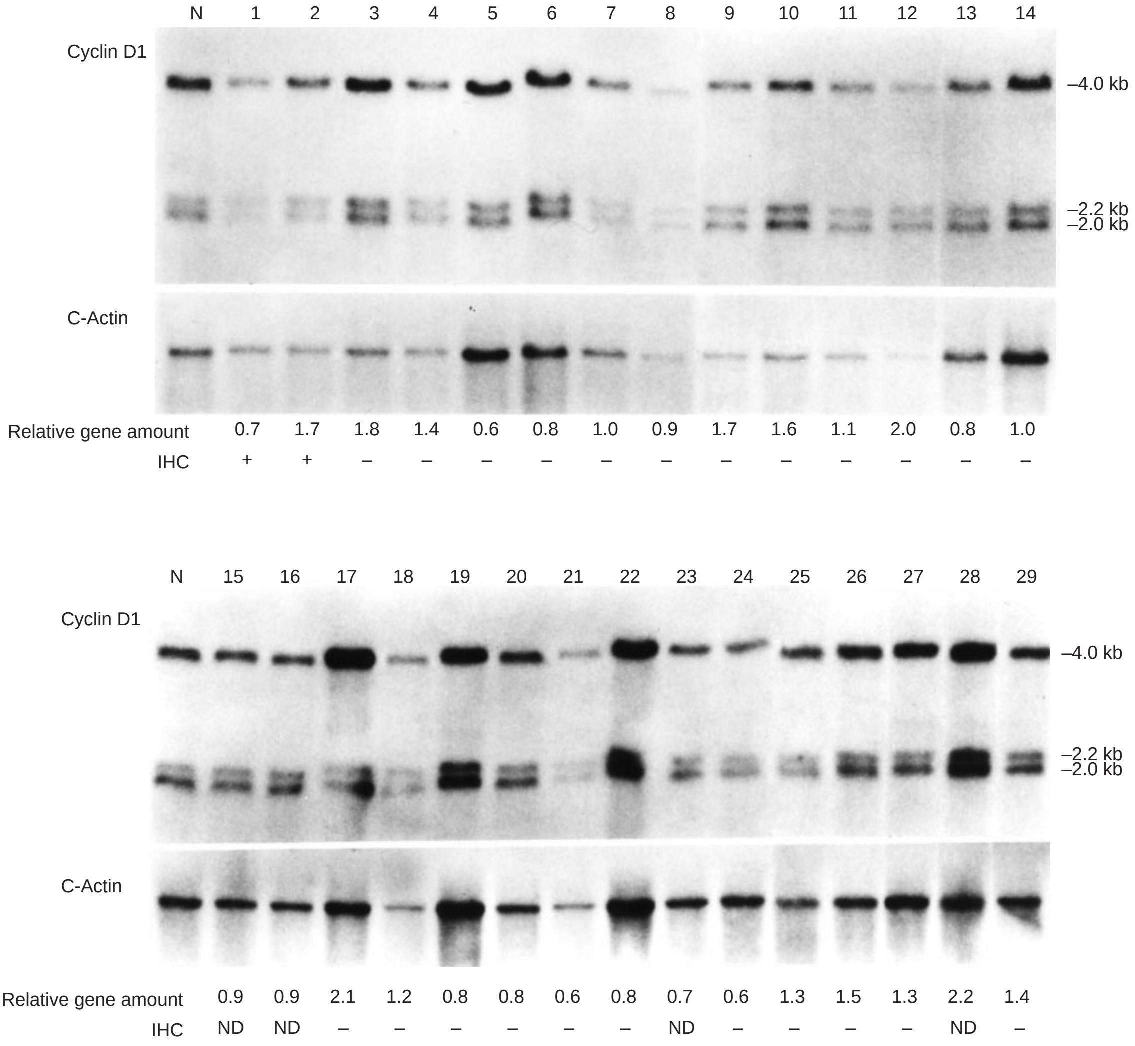

**Figure 2** Southern blot analysis of the cyclin D1 gene with *Eco*RI-digested genomic DNA from tumour samples (Lanes 1–29) and normal lung tissues (Lane N). Also shown is the internal control cardiac actin (C-Actin) band to which the cyclin D1 signal intensity was normalized for densitometer analysis. The relative cyclin D1 gene amount was defined as the normalized cyclin D1 signal intensity in the tumour samples relative to that in the normal lung control (= 1.0). Relative cyclin D1 gene amounts of 29 tumour samples ranged from 0.6 to 2.2. IHC: positive (+) or negative (–) nuclear staining for cyclin D1 determined by immunohistochemistry. ND: not determined

### DISCUSSION

The present study demonstrated expression of cyclin D1 without amplification of its gene in primary NSCLCs. Cyclin D1 expression was frequently found in tumours with altered p53 protein expression, and cyclin D1+ tumours showed higher proliferative activity determined by the Ki-67 index than cyclin D1– tumours. Furthermore, cyclin D1 expression was associated with a favourable clinical outcome. In addition, cyclin D1 expression was found in both p16/RB+ and p16/RB– NSCLCs in this study, results consistent with previous studies (Shapiro et al, 1995; Tanaka et al, 1998).

The frequency and subcellular localization of cyclin D1 expression determined by immunohistochemistry varied among studies including the present study (Betticher et al, 1996; Nishio et al, 1997). We found cyclin D1 expression in 13 (11.7%) of 111 NSCLCs invariably in nucleus of tumour cells. Betticher et al (1996) reported nuclear staining of cyclin D1 in ten (18.9%) of 53 NSCLCs and its cytoplasmic staining in 22 (41.5%) of 53 NSCLCs. On the other hand, nuclear staining of cyclin D1 was detected in 81 (38.9%) of 208 NSCLCs by Nishio et al (1997). Positive cytoplasmic staining in a few squamous cell carcinomas was not scored as positive staining in the study. These discrepancies of frequency and subcellular localization of cyclin D1 expression may be attributed not only to the difference of antibodies and antigen retrieval methods used, but also to the difference of fixation and preservation of materials: we used fresh, rapidly frozen tissues and fixed them in formaldehyde, although the two previous studies used archival formalin-fixed paraffin-embedded tissues.

In the absence of amplification and rearrangement of the cyclin D1 gene, mechanisms other than these may be responsible for

 © 1999 Cancer Research Campaign

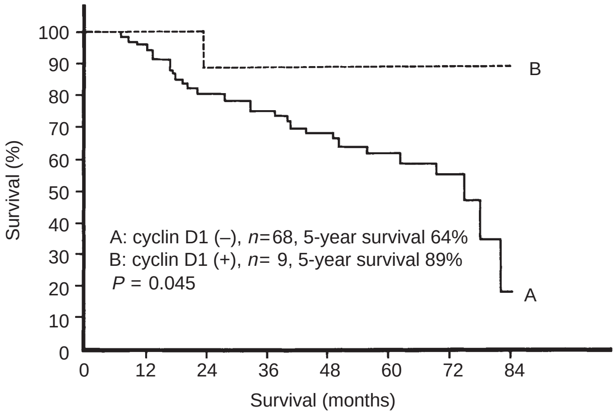

**Figure 3** Kaplan–Meier survival curves of NSCLC patients who underwent potentially curative resection. Survival curves of 77 such NSCLC patients are stratified by cyclin D1 expression

**Table 2** Ki-67 indices, and p53, p16 and RB protein expression in NSCLC groups divided by cyclin D1 expression

| | Cyclin D1 expression | | |
|---|---|---|---|
| **Characteristic** | **(–)** | **(+)** | ***P*** |
| Ki-67 index[a] | | | |
| Mean ± s.d. | 20.3 ± 22.2 | 26.2 ± 17.5 | 0.08 |
| Median | 12.0 | 25.0 | |
| Range | 1.0–92.0 | 1.8–64.6 | |
| p53[b] | | | |
| p53– | 60 | 4 | 0.04 |
| p53+ | 38 | 9 | |
| p16[c] | | | |
| p16+ | 69 | 11 | 0.5 |
| p16– | 27 | 2 | |
| RB[d] | | | |
| RB+ | 89 | 12 | 1.0 |
| RB– | 9 | 1 | |
| p16/RB[e] | | | |
| p16/RB+ | 60 | 10 | 0.4 |
| p16/RB– | 36 | 3 | |

[a]Determined for 91 cyclin D1– and 11 cyclin D1+ tumours. [b]p53– and p53+, tumours without and with altered p53 protein expression respectively. [c]p16+ and p16–, tumours retaining and lacking normal p16 protein expression respectively. [d]RB+ and RB–, tumours retaining and lacking normal RB protein expression respectively. [e]p16/RB+, tumours retaining both p16 and RB protein expression; p16/RB–, tumours lacking either p16 or RB protein expression.

**Table 3** Univariate analysis of potential prognostic factors in potentially curatively resected NSCLCs

| Characteristic | Hazard ratio | 95% Confidence interval | *P* |
|---|---|---|---|
| Age | 0.99 | 0.95–1.03 | 0.5 |
| Gender | 1.89 | 0.78–4.62 | 0.2 |
| Chemotherapy | 1.74 | 0.85–3.5 | 0.1 |
| Histological type[a] | 1.41 | 0.38–5.18 | 0.6 |
| pT classification[b] | 3.38 | 1.06–10.75 | 0.04 |
| pN classification[c] | 3.47 | 1.45–8.33 | 0.005 |
| pStage[d] | 3.88 | 1.73–8.70 | 0.001 |
| Cyclin D1 | 0.16 | 0.02–1.22 | 0.08 |

[a]Squamous cell carcinoma vs non-squamous cell carcinoma. [b]pT1 vs pT2–3. [c]pN0 vs pN1–2. [d]pStage I vs pStage II–IIIa.

cyclin D1 expression. Expression of cyclin D1 has been shown to be up-regulated by a complex mechanism involving RB and p53, and down-regulation can be caused by oncogenic proteins of transforming DNA viruses, including SV40 large T antigen and E6 and E7 proteins of the human papillomavirus, which are known to inactivate tumour suppressor genes such as RB and p53 (Lukas et al, 1994*b*; Müller et al, 1994; Chen et al, 1995; Spitkovsky et al, 1995; Del Sal et al, 1996; Marhin et al, 1996). In the present study, cyclin D1 expression was observed not only in tumours with unaltered p53 and/or RB protein status but also in tumours with altered expression of these proteins, suggesting that p53 and RB proteins might not necessarily be involved in the regulation of cyclin D1 expression in NSCLCs. On the other hand, alterations of the pathways regulated by polypeptide growth factors such as epidermal growth factor (EGF) (Ravitz et al, 1996; Zhu et al, 1996), and by the *ras* oncogene product may contribute to cyclin D1 expression (Filmus et al, 1994; Peeper et al, 1997). We determined expression of the *ras* oncogene product by immunohistochemistry in this cohort of NSCLCs, using our previously reported methods (Harada et al, 1992). No significant association was observed between cyclin D1 and *ras* oncogene expression (data not shown). However, the association of cyclin D1 expression with K-*ras* mutation and with EGF and EGF receptor expression in NSCLCs remains to be determined.

In the present study, we found altered p53 protein expression more frequently in cyclin D1+ tumours than in cyclin D1– tumours. Co-expression of cyclin D1 and altered p53 protein has been reported in uterine endometrial carcinomas (Nikaido et al, 1996). Similarly, Mineta et al (1997) reported a correlation between p53 mutations and cyclin D1 amplification in head and neck squamous cell carcinomas. These results, including ours, suggest that abnormalities of cyclin D1 and p53 may present simultaneously during the development and progression of cancer (Uchimura et al, 1996), which is thought to represent a multistep process involving the progressive accumulation of genetic alterations (Fearon and Vogelstein, 1990).

The association of cyclin D1 expression with a higher Ki-67 index indicated increased growth potential of cyclin D1+ tumours. Expression of cyclin D1 in early G1 is reported to promote cell cycle progression through the G1 phase (Jiang et al, 1993). Ki-67 protein appears during the transition from G0 to G1, and remains detectable for the remainder of the cell cycle and subsequent cycles (Gerdes et al, 1984). This increased growth potential of cyclin D1+ tumours may be reflected in more frequent expression of cyclin D1 in tumours with advanced pT status (pT2–4) than in pT1 tumours. Alternatively, frequent expression of cyclin D1 in tumours with advanced pT status may indicate that cyclin D1 expression is a relatively late event in the development and progression of NSCLCs.

Patients with cyclin D1+ NSCLCs showed longer survival periods than those with cyclin D1– NSCLCs, and cyclin D1 expression tended to be a favourable prognostic factor in this cohort of NSCLCs. These findings are similar to those reported by others (Betticher et al, 1996, 1997; Nishio et al, 1997). Cyclin D1 seems to play a role not only in cell cycle promotion at the G1 checkpoint but also in the control of apoptosis (Sofer-Levi et al,

© 1999 Cancer Research Campaign

1996) and growth suppresssion (Del Sal et al, 1996; Marhin et al, 1996). Such multifunctional properties of cyclin D1 may have influence on a better clinical outcome of patients with cyclin D1+ NSCLCs. The present cohort included a limited number of potentially curatively resected NSCLCs in pStages I to IIIa with relatively short periods of observation after surgery. A larger study that includes patients with homogeneous stages of disease in longer periods of observation is currently ongoing, and will determine whether cyclin D1 expression can predict the clinical outcome of resected NSCLC patients.

In conclusion, the present study suggests that cyclin D1 expression may be involved in the development and progression of NSCLCs, their high proliferative activity, and a favourable clinical outcome.

### ACKNOWLEDGEMENTS

The authors thank Dr T Hirata of National Sapporo Minami Hospital, Sapporo, Japan, for providing resected NSCLC specimens, and Dr M Fujita of the Department of Pathology, National Sapporo Hospital, Sapporo, Japan, for providing an oesophageal cancer specimen with cyclin D1 expression, which was used as a positive control for immunostaining of cyclin D1. We also thank Dr A Arnold of Massachusetts General Hospital, Boston, MA, USA, and Dr F Gunning of Stanford University and Veterans Affairs Medical Center, Palo Alto, CA, USA, for providing human cyclin D1 and cardiac actin cDNAs respectively.

### REFERENCES

Åkervall JA, Michalides RJAM, Mineta H, Balm A, Borg A, Dictor M, Jin Y, Loftus B, Mertens F and Wennerberg JP (1997) Amplification of cyclin D1 in squamous cell carcinoma of the head and neck and the prognostic value of chromosomal abnormalities and cyclin D1 overexpression. *Cancer* **79**: 380–389

Bartkova J, Lukas J, Strauss M and Bartek J (1994) Cell cycle-related variation and tissue-restricted expression of human cyclin D1 protein. *J Pathol* **172**: 237–245

Beahrs OH, Henson DE, Hutter RVP and Kennedy BJ (1992) Lung. In: *Manual for Staging of Cancer*, 4th edn. American Joint Committee on Cancer: Chicago: 115–122

Betticher DC, Heighway J, Hasleton PS, Altermatt HJ, Ryder WDJ, Cerny T and Thatcher N (1996) Prognostic significance of CCND1 (cyclin D1) overexpression in primary resected non-small-cell lung cancer. *Br J Cancer* **73**: 294–300

Betticher DC, White GRM, Vonlanthen S, Liu X, Kappeler A, Altermatt H, Thatcher N and Heighway J (1997) G1 control gene status is frequently altered in resectable non-small-cell lung cancer. *Int J Cancer* **74**: 556–562

Buckley MF, Sweeney KJE, Hamilton JA, Sini RL, Manning DL, Nicholson RI, deFazio A, Watts CKW, Musgrove EA and Sutherland RL (1993) Expression and amplification of cyclin genes in human breast cancer. *Oncogene* **8**: 2127–2133

Chen X, Bargonetti J and Prives C (1995) p53, through p21 (WAF1/CIP1), induces cyclin D1 synthesis. *Cancer Res* **55**: 4257–4263

Del Sal G, Murphy M, Ruaro E, Lazarevic D, Levine A and Schneider C (1996) Cyclin D1 and p21/waf1 are involved in p53 growth suppression. *Oncogene* **12**: 177–185

Fearon ER and Vogelstein B (1990) A genetic model for colorectal tumorigenesis. *Cell* **61**: 759–767

Filmus J, Robles AI, Shi W, Wong MJ, Colombo LL and Conti C (1994) Induction of cyclin D1 overexpression by activated *ras*. *Oncogene* **9**: 3627–3633

Gerdes J, Lemke H, Baisch H, Wacker H, Schwab U and Stein H (1984) Cell cycle analysis of a cell proliferation-associated human nuclear antigen defined by the monoclonal antibody Ki-67. *J Immunol* **133**: 1710–1715

Hannon GJ and Beach D (1994) $p15^{INK4B}$ is a potential effector of TGF-β-induced cell cycle arrest. *Nature* **371**: 257–261

Harada M, Dosaka-Akita H, Miyamoto H, Kuzumaki N and Kawakami Y (1992) Prognostic significance of the expression of ras oncogene product in non-small-cell lung cancer. *Cancer* **69**: 72–77

Hinds PW, Dowdy SF, Eaton EN, Arnold A and Weinberg RA (1994) Function of a human cyclin gene as an oncogene. *Proc Natl Acad Sci USA* **91**: 709–713

Jiang W, Tomita N, Zhang Y, Lu S and Weinstein B (1992) Amplification and expression of the human cyclin D gene in esophageal cancer. *Cancer Res* **52**: 2980–2983

Jiang W, Kahn SM, Zhou P, Zhang Y, Cacace AM, Infante AS, Doi S, Santella RM and Weinstein IB (1993) Overexpression of cyclin D1 in rat fibroblasts causes abnormalities in growth control, cell cycle progression and gene expression. *Oncogene* **8**: 3447–3457

Kinoshita I, Dosaka-Akita H, Mishina T, Akie K, Nishi M, Hiroumi H, Hommura F and Kawakami Y (1996) Altered $p16^{INK4}$ and retinoblastoma protein status in non-small-cell lung cancer: potential synergistic effect with altered p53 protein on proliferative activity. *Cancer Res* **56**: 5557–5562

Lovec H, Sewing A, Lucibello FC, Müller R and Möröy T (1994) Oncogenic activity of cyclin D1 revealed through cooperation with Ha-*ras*: link between cell cycle control and malignant transformation. *Oncogene* **9**: 323–326

Lukas J, Pagano M, Staskova Z, Draetta G and Bartek J (1994*a*) Cyclin D1 protein oscillates and is essential for cell cycle progression in human tumour cell lines. *Oncogene* **9**: 707–718

Lukas J, Müller H, Bartkova J, Spitkovsky D, Kjerulff AA, Jansen-Durr P, Strauss M and Bartek J (1994*b*) DNA tumor virus oncoproteins and retinoblastoma gene mutations share the ability to relieve the cell's requirement for cyclin D1 function in G1. *J Cell Biol* **125**: 625–638

Lukas J, Parrr D, Aagaard L, Mann DJ, Bartkova J, Strauss M, Peters G and Bartek J (1995) Retinoblastoma-protein dependent cell-cycle inhibition by the tumour suppressor p16. *Nature* **375**: 503–506

McIntosh GG, Anderson JJ, Milton I, Steward M, Parr AH, Thomas MD, Henry JA, Angus B, Lennard TWJ and Horne CHW (1995) Determination of the prognostic value of cyclin D1 overexpression in breast cancer. *Oncogene* **11**: 885–891

Marchetti A, Doglioni C, Barbareschi M, Buttitta F, Pellegrini S, Gaeta P, La Rocca R, Merlo G, Chella A, Angeletti CA, Palma PD and Bevilacqua G (1998) Cyclin D1 and retinoblastoma susceptibility gene alterations in non-small-cell lung cancer. *Int J Cancer* **75**: 187–192

Marhin WW, Hei Y, Chen S, Jiang Z, Gallie BL, Phillips RA and Penn LZ (1996) Loss of Rb and myc activation co-operate to suppress cyclin D1 and contribute to transformation. *Oncogene* **12**: 43–52

Mate JL, Ariza A, Aracil C, Lopez D, Isamat M, Perez-Piteira J and Navas-Palacios JJ (1996) Cyclin D1 overexpression in non-small-cell lung carcinoma: correlation with Ki-67 labelling index and poor cytoplasmic differentiation. *J Pathol* **180**: 395–399

Matsushime H, Roussel MF, Ashmun RA and Sherr CJ (1991) Colony-stimulating factor 1 regulates novel cyclins during the G1 phase of the cell cycle. *Cell* **65**: 701–713

Mineta H, Borg Å, Dictor M, Wahlberg P and Wennerberg J (1997) Correlation between p53 mutation and cyclin D1 amplification in head and neck squamous cell carcinoma. *Oral Oncol* **33**: 42–46

Motokura T, Bloom T, Kim HG, Juppner H, Ruderman JV, Kronenberg HM and Arnold A (1991) A novel cyclin encoded by a bcl-1-linked candidate oncogene. *Nature* **350**: 512–515

Müller H, Lukas J, Schneider A, Warthoe P, Bartek J, Eilers M and Strauss M (1994) Cyclin D1 expression is regulated by the retinoblastoma protein. *Proc Natl Acad Sci USA* **91**: 2945–2949

Naitoh H, Shibata J, Kawaguchi A, Kodama M and Hattori T (1995) Overexpression and localization of cyclin D1 mRNA and antigen in esophageal cancer. *Am J Pathol* **146**: 1161–1169

Nakagawa H, Wang TC, Zukerberg L, Odze R, Togawa K, May GHW, Wilson J and Rustgi AK (1997) The targeting of the cyclin D1 oncogene by an Epstein–Barr virus promoter in transgenic mice causes dysplasia in the tongue, esophagus and forestomach. *Oncogene* **14**: 1185–1190

Nikaido T, Li S, Shiozawa T and Fujii S (1996) Coabnormal expression of cyclin D1 and p53 protein in human uterine endometrial carcinomas. *Cancer* **78**: 1248–1253

Nishida N, Fukuda Y, Komeda T, Kita R, Sando T, Furukawa M, Amenomori M, Shibagaki I, Nakao K, Ikenaga M and Ishizaki K (1994) Amplification and overexpression of the cyclin D1 gene in aggressive human hepatocellular carcinoma. *Cancer Res* **54**: 3107–3110

Nishio M, Koshikawa T, Yatabe Y, Kuroishi T, Suyama M, Nagatake M, Sugiura T, Ariyoshi Y, Mitsudomi T, Takahashi T and Takahashi TA (1997) Prognostic significance of cyclin D1 and retinoblastoma expression in combination with p53 abnormalities in primary, resected non-small-cell lung cancers. *Clin Cancer Res* **3**: 1051–1058

 *© 1999 Cancer Research Campaign*

Peeper DS, Upton TM, Ladha MH, Neuman E, Zalvide J, Bernards R, DeCaprio JA and Ewen M (1997) Ras signalling linked to the cell-cycle machinery by the retinoblastoma protein. *Nature* **386**: 177–181

Ravitz MJ, Yan S, Dolce C, Kinniburgh AJ and Wenner CE (1996) Differential regulation of p27 and cyclin D1 by TGF-β and EGF in C3H 10T1/2 mouse fibroblasts. *J Cell Physiol* **168**: 510–520

Serrano M, Hannon GJ and Beach D (1993) A new regulatory motif in cell-cycle control causing specific inhibition of cyclin D/CDK4. *Nature* **366**: 704–707

Shapiro GI, Edwards CD, Kobzik L, Godleski J, Richards W, Sugarbaker DJ and Rollins BJ (1995) Reciprocal Rb inactivation and $p16^{INK4}$ expression in primary lung cancers and cell lines. *Cancer Res* **55**: 505–509

Sherr CJ (1996) Cancer cell cycles. *Science* **274**: 1672–1677

Sofer-Levi Y and Resnitzky D (1996) Apoptosis induced by ectopic expression of cyclin D1 but not cyclin E. *Oncogene* **13**: 2431–2437

Spitkovsky D, Steiner, Gopalkrishnan RV, Eilers M and Jansen-Dürr P (1995) The role of p53 in coordinated regulation of cyclin D1 and p21 gene expression by the adenovirus E1A and E1B oncogenes. *Oncogene* **10**: 2421–2425

Tanaka H, Fujii Y, Hirabayashi H, Miyoshi S, Sakaguchi M, Yoon H and Matsuda H (1998) Disruption of the RB pathway and cell-proliferative activity in non-small-cell lung cancers. *Int J Cancer* **79**: 111–115

Uchimura K, Endo K, Fujinuma H, Zukerberg L, Arnold A and Motokura T (1996) Oncogenic collaboration of the cyclin D1 (PRAD1, bcl-1) gene with a mutated p53 and an activated ras oncogene in neoplastic transformation. *Jpn J Cancer Res* **87**: 459–465

Wang TC, Cardiff RD, Zukerberg L, Lees E, Arnold A and Schmidt EV (1994) Mammary hyperplasia and carcinoma in MMTV-cyclin D1 transgenic mice. *Nature* **369**: 669–671

Weinberg RA (1995) The retinoblastoma protein and cell cycle control. *Cell* **81**: 323–330

WHO (1982) *Histological Typing of Lung Tumors*, 2nd edn. pp. 25–26. World Health Organization: Geneva

Zhu X, Ohtsubo M, Böhmer RM, Roberts JM and Assoian RK (1996) Adhesion-dependent cell cycle progression linked to the expression of cyclin D1, activation of cyclin E-cdk2, and phosphorylation of the retinoblastoma protein. *J Cell Biol* **133**: 391–403

*© 1999 Cancer Research Campaign*
